# Urinary biomarker discovery in gliomas using mass spectrometry-based clinical proteomics

**DOI:** 10.1186/s41016-020-00190-5

**Published:** 2020-04-14

**Authors:** Jianqiang Wu, Jun Zhang, Jing Wei, Yuanli Zhao, Youhe Gao

**Affiliations:** 1 Medical Research Center, Peking Union Medical College Hospital, Chinese Academy of Medical Sciences & Peking Union Medical College, Beijing, 100730 China; 2 Department of Neurosurgery, Peking University International Hospital, Peking University, Beijing, 102206 China; 3 Department of Biochemistry, Gene Engineering Drug and Biotechnology Beijing Key Laboratory, School of Life Sciences, Beijing Normal University, No.19 Xinjiekouwai Street, Beijing, 100875 China; 4 Department of Neurosurgery, Beijing Tian Tan Hospital, Capital Medical University; China National Clinical Research Center for Neurological Diseases, Beijing, 100050 China

**Keywords:** Glioma, Biomarkers, Urine, Proteomics

## Abstract

**Background:**

Gliomas are the most common primary malignant brain tumors and have a poor prognosis. Early detection of gliomas is crucial to improve patient outcomes. Urine accumulates systematic body changes and thus serves as an excellent early biomarker source.

**Methods:**

At the biomarker discovery phase, we performed a self-controlled proteomics analysis by comparing urine samples collected from five glioma patients at the time of tumor diagnosis and after surgical removal of the tumor. At the biomarker validation phase, we further validated some promising proteins using parallel reaction monitoring (PRM)-based targeted proteomics in another cohort, including glioma, meningioma, and moyamoya disease patients as well as healthy controls.

**Results:**

Using label-free proteome quantitation (LFQ), we identified twenty-seven urinary proteins that were significantly changed after tumor resection, many of which have been previously associated with gliomas. The functions of these proteins were significantly enriched in the autophagy and angiogenesis, which are associated with glioma development. After targeted proteomics validation, we identified a biomarker panel (AACT, TSP4, MDHM, CALR, LEG1, and AHSG) with an area under the curve (AUC) value of 0.958 for the detection of gliomas. Interestingly, AACT, LEG1, and AHSG are also potential cerebrospinal fluid or blood biomarkers of gliomas.

**Conclusions:**

Using LFQ and PRM proteome quantification, we identified candidate urinary protein biomarkers with the potential to detect gliomas. This study will also provide clues for future biomarker studies involving brain diseases.

## Background

Gliomas are the most common type of primary brain tumor in adults, representing 81% of malignant brain tumors [1]. Malignant gliomas have a very poor prognosis and a high risk of tumor recurrence after treatment. Early detection of gliomas and disease monitoring for tumor recurrence is essential to improve patient survival. However, no general screening protocols are currently available to reveal asymptomatic brain tumors [2]. Thus, there is an urgent need to discover noninvasive biomarkers for tumor detection in glioma patients.

Biomarkers are measurable changes associated with physiological or pathophysiological processes [3]. Without homeostatic control, urine accumulates systematic changes in the body and thus is an ideal biomarker source, with the potential to reflect small, early pathological changes [4]. In addition, urine can be collected noninvasively in large volumes. With the rapid development of mass spectrometry (MS) techniques, urinary proteomics has become a popular field in biomarker discovery. The urinary proteome contains both plasma proteins filtered by the glomeruli and proteins shed by cells within the urogenital system. Therefore, changes in the urine proteome can reflect pathological conditions of the whole body. To date, more than 5000 proteins have been identified through deep profiling of the normal human urinary proteome [5]. Despite these advantages, urinary proteomics has been underutilized in brain disorders compared with its wide application in renal diseases. Urine is located far from the brain; thus, there may be doubts as to whether pathological changes in the brain will be detected in the urine. However, there is an emerging evidence that changes in urinary proteins could reflect brain disorders [2, 6, 7]. In our previous study, it was also observed that changes in the urine proteome occurred before any changes were visible on brain imaging in a rat model of glioblastoma multiforme (GBM) [8].

In animal models, the effects of genetic and environmental factors on the urine proteome are minimal, which is useful for identifying disease biomarkers [9]. However, the urine proteome of patients may be affected by some factors, such as gender, aging, exercise, and hormone conditions [4, 10–13]. Because a self-controlled study can avoid the interference of individual differences among patients, we speculate that promising glioma biomarkers can be identified from a relatively small number of glioma patient urine samples taken before and after tumor resection.

In this study, potential urinary biomarkers of gliomas patients were discovered using both label-free quantification (LFQ) and parallel reaction monitoring (PRM)-based targeted quantification. Finally, a biomarker panel consisting of six differential proteins was identified with a good diagnostic performance for the detection of gliomas.

## Methods

### Subjects and study design

All patients and healthy controls were recruited from the Peking University International Hospital. Glioma patients without hypertension, diabetes, or other tumor diseases were enrolled in this study, and these patients have primary gliomas. The final tumor diagnosis was based on the histopathology of resected tissues. All subjects in this experiment were informed of the purpose of the study and signed informed consent forms before inclusion. The study was approved by the local ethical committee of Peking University International Hospital (no. 2018029).

In the biomarker discovery phase of this study, urine samples from five glioma patients were collected at the time of tumor diagnosis and after surgical removal of the tumors. A comparative proteomic analysis of urine samples before and after tumor resection was performed using label-free quantitation (LFQ). In the biomarker validation phase of this study, some candidate proteins were selected both from LFQ and blood or cerebrospinal fluid (CSF) biomarkers of glioma in previous studies. These proteins were further validated using parallel reaction monitoring (PRM)-based targeted proteome quantification in urine samples of another cohort, which included 10 glioma patients, 5 meningioma patients, 6 moyamoya patients, and 12 healthy controls.

### Urine collection and sample preparation

Midstream first morning urine was collected from all subjects. In the biomarker discovery phase, preoperative urine samples were collected from glioma patients one day before surgery. After surgical treatment, these patients received 40 mg methylprednisolone (9 am and 3 pm) for 5-6 days. Postoperative urine samples were collected 1-2 weeks after surgery and just before discharge to prevent trauma effects and drugs from affecting the urine proteome. In the validation phase, urine samples of patients were all collected before surgical treatment.

After urine collection, samples were immediately centrifuged at 5000×*g* for 20 min. Urinary proteins were extracted from the supernatant with ethanol at − 20 °C overnight, followed by centrifugation at 12,000×*g* for 20 min. The precipitate was then resuspended in lysis buffer (8 M urea, 2 M thiourea, 50 mM Tris, and 25 mM DTT). The urinary proteins were prepared using the filter-aided sample preparation method for proteomics analysis [14]. Briefly, 100 μg of urinary protein from each individual sample was denatured with 20 mM dithiothreitol at 37 °C for 1 h and alkylated with 50 mM iodoacetamide in the dark for 30 min. The samples were then loaded onto filter devices with a cut-off of 10 kDa and centrifuged at 14,000×*g* at 18 °C. Then, after being washed twice with 8 M urea (pH 8.5) and 25 mM NH_4_HCO_3_, the samples were redissolved in 25 mM NH_4_HCO_3_ and digested with trypsin at 37 °C overnight. Finally, the peptide mixtures were desalted using OASIS HLB 1 cc/10 mg solid-phase extraction cartridges (Waters, Milford, MA) and dried by vacuum evaporation.

### Label-free proteome quantitation using LC-MS/MS

Digested peptide samples were redissolved in 0.1% formic acid. For proteomics analysis, 1 μg of peptides from an individual sample was loaded onto a trap column and separated on a reverse-phase C18 analytical column using the EASY-nLC 1200 HPLC system (Thermo Fisher Scientific, Waltham, MA, USA). Then, the peptides were analyzed with an Orbitrap Fusion Lumos Tribrid mass spectrometer (Thermo Fisher Scientific, Waltham, MA, USA). The elution for the analytical column was carried out over 60 min at a flow rate of 300 nL/min. The mass spectrometer was set in positive ion mode and operated in data-dependent acquisition mode, with full MS scanning from 150 to 2000 m/z with resolution at 120,000 and MS/MS scanning from 110 to 2000 m/z with resolution at 30,000 in an Orbitrap. MS data were acquired in high-sensitivity mode, and 30% higher-energy collisional dissociation (HCD) energy and charge-state screening (+ 2 to + 7) were used. To identify more urinary proteins and validate the differentially expressed proteins (DEPs) identified above, we reanalyzed the ten peptide samples using a 90 min elution time, keeping the other MS parameters the same as above.

The proteomic data were searched against the Swiss-Prot Human database (released in 2017, containing 20,169 sequences) using the Mascot software (version 2.6.1, Matrix Science, London, UK). The parent ion tolerance was set at 10 ppm, and the fragment ion mass tolerance was set to 0.05 Da. Up to two missed cleavage sites in the trypsin digestion were allowed. Carbamidomethylation of cysteines was set as a fixed modification, and the oxidation of methionine was considered a variable modification. LFQ was performed using the Scaffold software (version 4.8.4, Proteome Software Inc., Portland, OR). Peptide identifications were accepted when we detected ≥ 2 unique peptides at a 1.0% false discovery rate (FDR) using the Scaffold Local FDR algorithm. The total spectra accounts of these ten samples were normalized using the Scaffold software. Spectral counting was used to compare the protein abundance between groups as previously described [15, 16].

### Targeted validation using PRM-based proteomics quantification

First, pooled peptide samples (2 μg of each sample) were subjected to LC-MS/MS analysis with six technical replicates. The Skyline software (version 3.6.1) was used to build the spectrum library and screen peptides for PRM analysis [17]. For each targeted protein, two to six peptides were selected using the following criteria: identified in the untargeted analysis with *q* value < 1%, completely digested by trypsin, 8-18 amino acid residues, exclusion of the first 25 N-terminal amino acids, and carbamidomethylation of cysteine as the fixed modification. Only unique peptides of each protein were used for PRM quantitation. The retention time (RT) segment was set to ± 2 min for each targeted peptide, with its expected RT in the center based on the pooled sample analysis. After further optimization, 48 proteins were ultimately used for validation by PRM-based targeted proteomics. The technical reproducibility of the PRM assay was assessed, and 191 targeted peptides had CV values less than 20%.

All of the MS data were further processed with Skyline. The transition settings in Skyline were as follows: precursor charges, + 2 to + 4; ion types, b, y, p; the product ions from ion 3 to last ion − 1; ion match tolerance, 0.02 m/z; six most intense product ions were picked, and the variable “min dotp” was set to 0.7. Each protein was quantitated by summing the fragment areas from its corresponding transitions. Prior to the statistical analysis, the summation of fragment area was performed by log_2_ transformation. The differential proteins were identified using Kruskal-Wallis and multiple comparisons, and significance was set at a *P* value < 0.05.

## Results

### Clinical characteristics of subjects

In the biomarker discovery phase, a total of 5 consecutive histopathologically diagnosed glioma patients were recruited, including 4 glioma patients with WHO-IV grade and 1 glioma patients with WHO-II grade. Images of a representative patient brain before and after tumor resection are shown in Additional file: Fig. S1. In the biomarker validation phase, another 10 glioma patients, 11 disease controls (5 meningioma patients and 6 moyamoya disease patients), and 12 healthy controls were enrolled. These glioma patients included 2 glioma patients with WHO-II grade, 2 glioma patients with WHO-III grade, and 6 glioma patients with WHO-IV grade. Subjects in each group were sex and age matched. Glioma patients in this study were classified according to their WHO grades (I-IV). The clinical characteristics of the subjects are summarized in Table 1.

**Table 1:** Clinical characteristics of subjects in this study

Group	***N***	Sex (M/F)	Age	Histology^**#**^
**Discovery phase**				
Gliomas	5	2/3	45.20 ± 10.50	WHO-II (1); WHO-IV (4)
**Validation phase**				
Gliomas	10	6/4	44.78 ± 12.20	WHO-II (2); WHO-III (2); WHO-IV(6)
Meningioma	5	2/3	54.60 ± 9.42	--
Moyamoya	6	3/3	37.67 ± 9.69	--
Healthy control	12	5/7	42.17 ± 8.42	--

Glioma grade classified as World Health Organization (WHO) grades I–IV

*M* male; *F* female

### Changes in the urine proteome after tumor resection

**Fig. 1:**
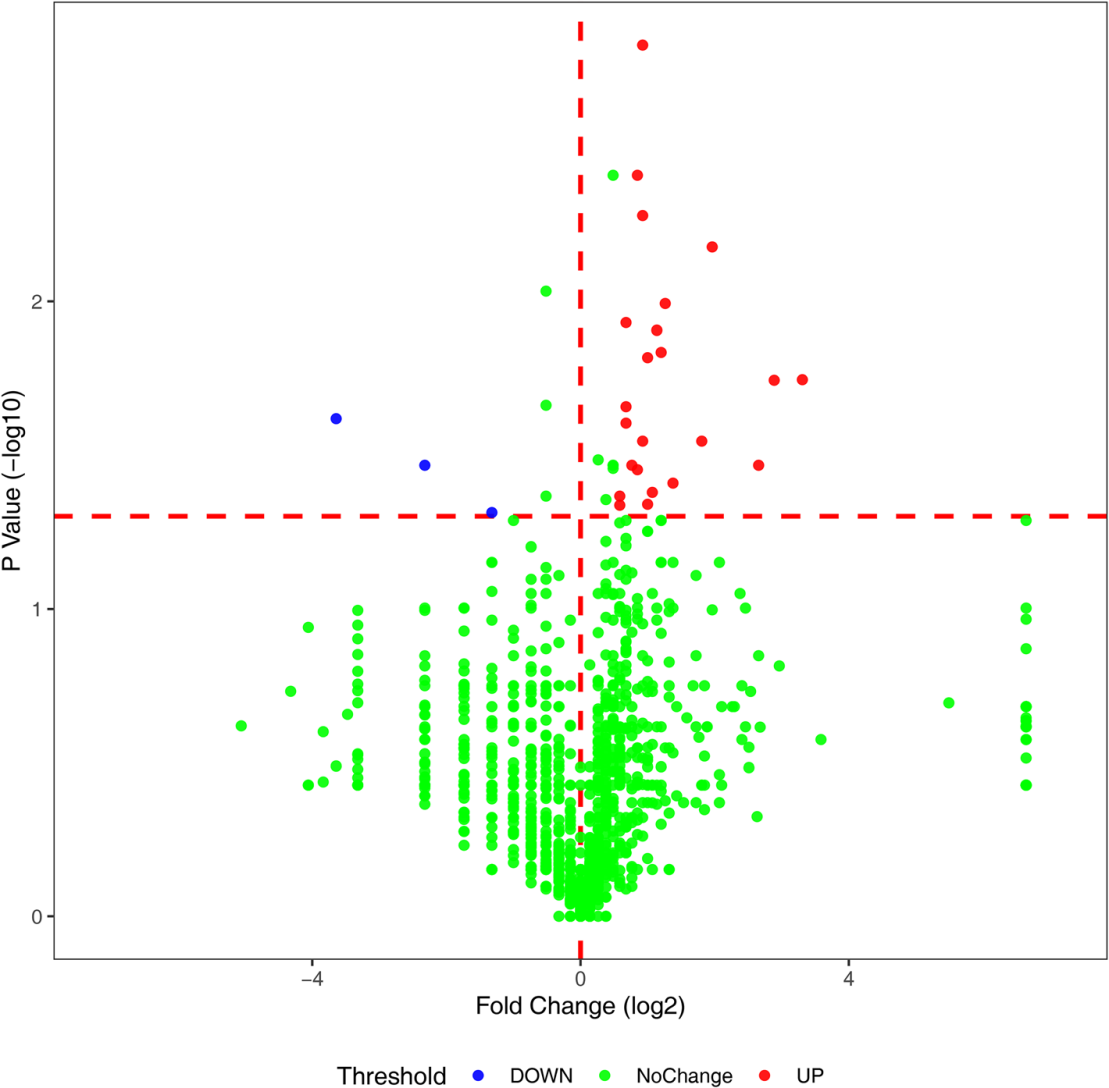
Volcano plots of DEPs by comparing postoperative urine samples with preoperative urine samples

In the first LFQ analysis, a total of 1377 urinary proteins with ≥ 2 unique peptides were identified at a protein identification FDR of < 1%. Urine samples were divided into a patient group (before tumor resection) and a treated group (after tumor resection). Differentially expressed proteins (DEPs) were screened with the following criteria: fold change ≥ 1.50 between the two groups and *P* value < 0.05 using a paired *t* test or Wilcoxon test; all five patient samples exhibited similar changes in protein levels after surgical treatment. The volcano plot of differential proteins are shown in Fig. 1. Although the *P* values of CALR and CEACAM1 were not significant, their fold changes were infinite. They were also considered as DEPs for further validation. Ultimately, a total of 27 DEPs were identified; details on these proteins are described in Table 2. Interestingly, 16 of those DEPs had been reported to be associated with gliomas in previous studies. To identify more urinary proteins and validate the alterations in protein levels, we reanalyzed the ten peptide samples with an extended elution time of 90 min. A total of 1652 urinary proteins were identified in this proteomic analysis. Of the 27 DEPs, 17 proteins were verified to also have significantly different levels in this analysis (Table 2).

**Table 2:** Differential proteins identified in urine samples before and after tumor resection in glioma patients

Uniprot	Description	FC^a^	P	Ref^b^
P35443	Thrombospondin-4 (TSP4)^#^	↓ 13.0	0.024	
P61604	10 kDa heat shock protein, mitochondrial (CH10)^#^	↑ 8.5	0.018	
Q8IZF2	Adhesion G protein-coupled receptor F5 (AGRF5)^#^	↑ 7.0	0.018	
P20073	Annexin A7 (ANXA7)	↑ 6.0	0.034	[18]
P40926	Malate dehydrogenase, mitochondrial (MDHM)^#^	↑ 4.2	0.007	
P02511	Alpha-crystallin B chain (CRYAB)	↑ 3.9	0.033	[19]
P09417	Dihydropteridine reductase (DHPR)	↑ 2.5	0.039	[20]
O43451	Maltase-glucoamylase, intestinal (MGA)^#^	↑ 2.4	0.010	
P13489	Ribonuclease inhibitor (RINI)^#^	↓ 2.4	0.049	
Q9H3G5	Probable serine carboxypeptidase (CPVL)^#^	↑ 2.3	0.015	
P13473	Lysosome-associated membrane glycoprotein 2 (LAMP2)^#^	↑ 2.2	0.012	[21]
Q92820	Gamma-glutamyl hydrolase (GGH)	↑ 2.0	0.015	
Q07075	Glutamyl aminopeptidase (AMPE)^#^	↑ 2.0	0.046	[20]
Q5ZPR3	CD276 antigen (CD276)^#^	↑ 1.9	0.042	[22]
O96009	Napsin-A (NAPSA)	↑ 1.9	0.005	
Q13510	Acid ceramidase (ASAH1)^#^	↑ 1.9	0.001	[23]
Q9BQ51	Programmed cell death 1 ligand 2 (PD1L2)	↑ 1.9	0.004	[24, 25]
P11279	Lysosome-associated membrane glycoprotein 1 (LAMP1)^#^	↑ 1.8	0.028	[20]
Q13228	Selenium-binding protein 1 (SBP1)^#^	↑ 1.7	0.035	[26]
P42785	Lysosomal Pro-X carboxypeptidase (PCP)^#^	↑ 1.6	0.012	
P08236	Beta-glucuronidase (BGLR)	↑ 1.6	0.022	[27]
P15144	Aminopeptidase N (AMPN)^#^	↑ 1.6	0.043	[28]
P01833	Polymeric immunoglobulin receptor (PIGR)	↑ 1.5	0.043	[29]
Q96PD5	N-acetylmuramoyl-L-alanine amidase (PGRP2) ^#^	↓ 1.5	0.043	
P07339	Cathepsin D (CTSD)	↑ 1.5	0.043	[30]
P13688	Carcinoembryonic antigen-related cell adhesion molecule 1 (CEACAM1)^#^	↑ ∞	0.052	[31]
P27797	Calreticulin (CALR)	↓ ∞	0.068	[32]

^**#**^Differential urinary proteins from both proteome analyses are in bold

^a^Change trends of proteins after tumor resection

^b^Proteins associated with gliomas in previous studies

### Functional analysis of differential urinary proteins after tumor resection

**Fig. 2:**
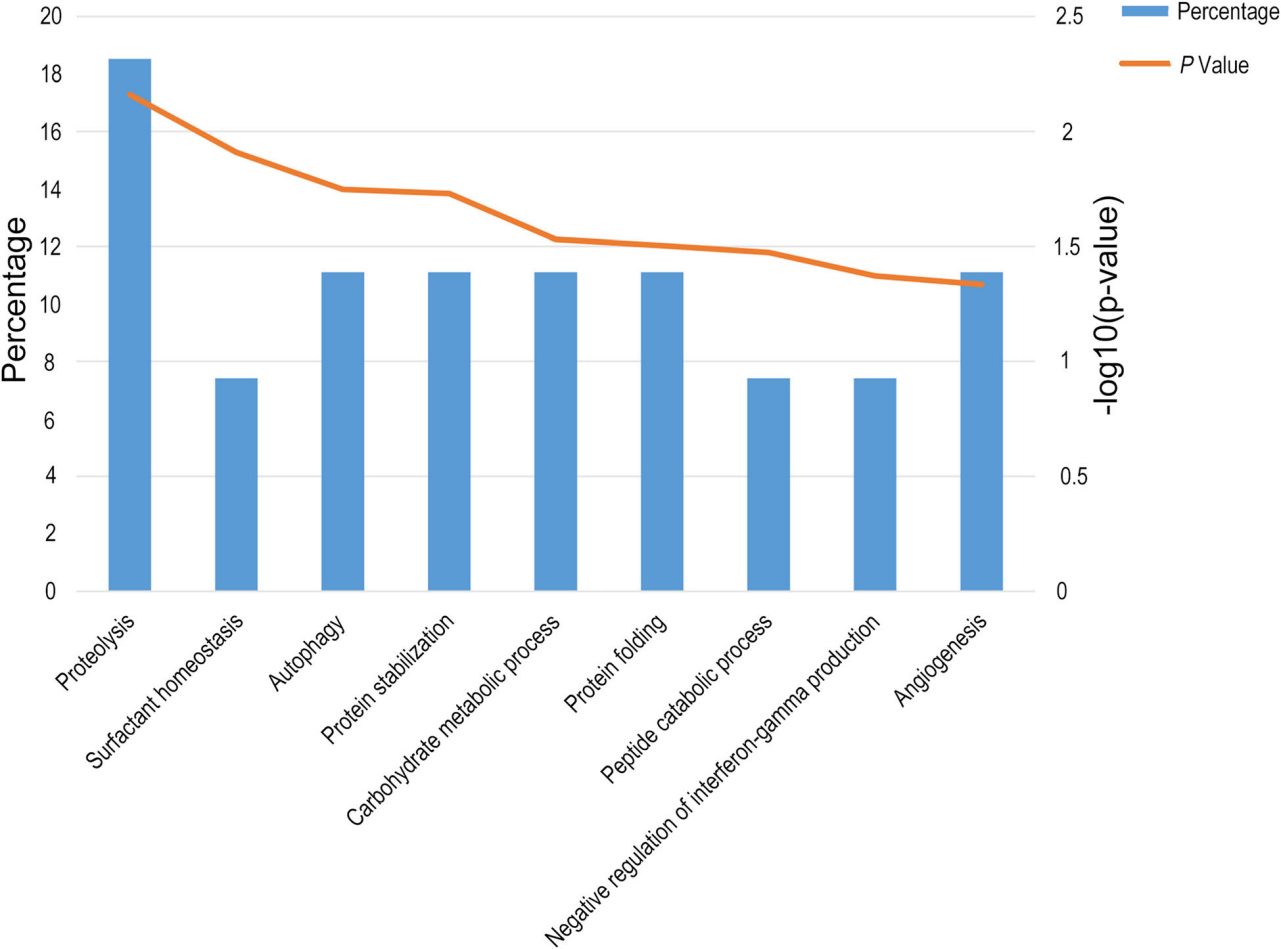
Functional annotation of differential urinary proteins before and after tumor resection

Functional annotation of the DEPs was performed using the Database for Annotation, Visualization, and Integrated Discovery (DAVID); they were classified as belonging a biological process, molecular function, or cellular component. In the biological process category, the terms *regulation of tissue remodeling*, *autophagy*, *negative regulation of gene expression*, *positive regulation of natural killer cell-mediated cytotoxicity,* and *angiogenesis* were overrepresented (Fig. 2). In the cellular component category, the majority of the identified proteins bore the term *extracellular exosome*, *lysosome*, *extracellular space,* or *membrane protein*. In the molecular function category, the terms *peptide binding*, *integrin binding,* and *metalloaminopeptidase activity* were overrepresented. After these categories were determined, the Kyoto Encyclopedia of Genes and Genomes (KEGG) was used for pathway enrichment analysis of the DEPs. The pathways for the lysosome, the renin-angiotensin system, the phagosome, autophagy, and folate biosynthesis were significantly enriched.

### Validation of candidate urine proteins using PRM-based targeted proteomics

**Fig. 3:**
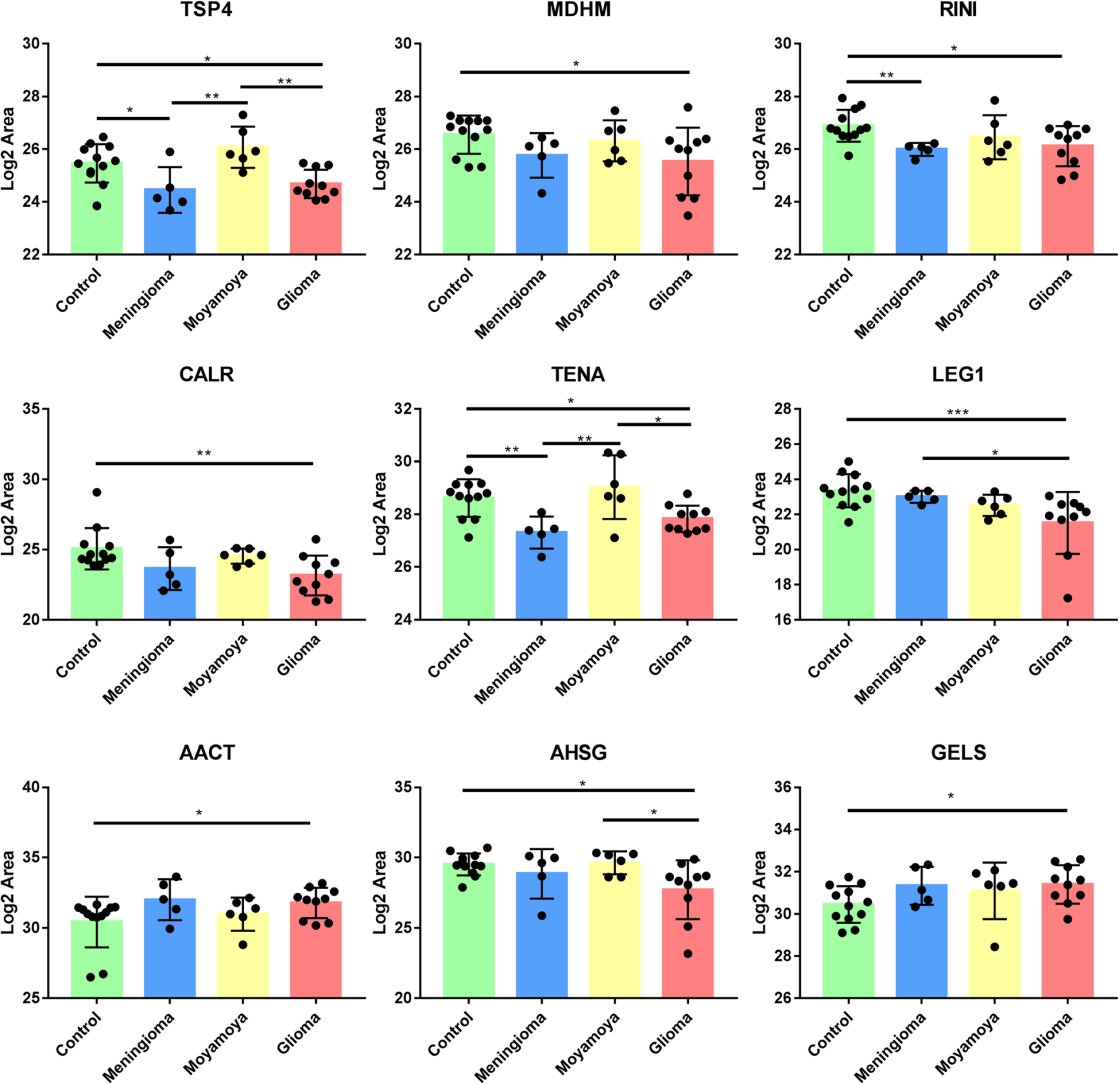
Targeted quantification of candidate urine biomarkers for gliomas detection

In addition to DEPs identified by LFQ experiments, 27 promising proteins that were previously reported as blood or CSF biomarker candidates of gliomas were also used for PRM analysis. The details of these 27 biomarker candidates are listed in Additional file: Table S1. After PRM optimization and screening, 46 proteins were ultimately quantified. By PRM-based quantification, nine urinary proteins showed significant differences between glioma patients and healthy controls, including TSP4, MDHM, RINI, CALR, TENA, LEG1, AACT, AHSG, and GELS (Fig. 3). A total of ten urine proteins showed significant differences between meningioma patients and healthy controls, including TSP1, TSP4, CH10, RINI, LAMP1, LAMP2, SBP1, CRYAB, TENA, and ITM2B. Because only glioma-related proteins were used for targeted quantification, only the GGH and NCAM1 proteins showed differential abundance between moyamoya disease patients and healthy controls. Interestingly, seven urinary proteins were differentially abundant between glioma and meningioma patients, including AMP4, CD276, LAMP1, NAPSA, LEG1, DNAS1, and BGAL. This result suggests that urine proteins could be used to distinguish different brain tumors.

**Fig. 4:**
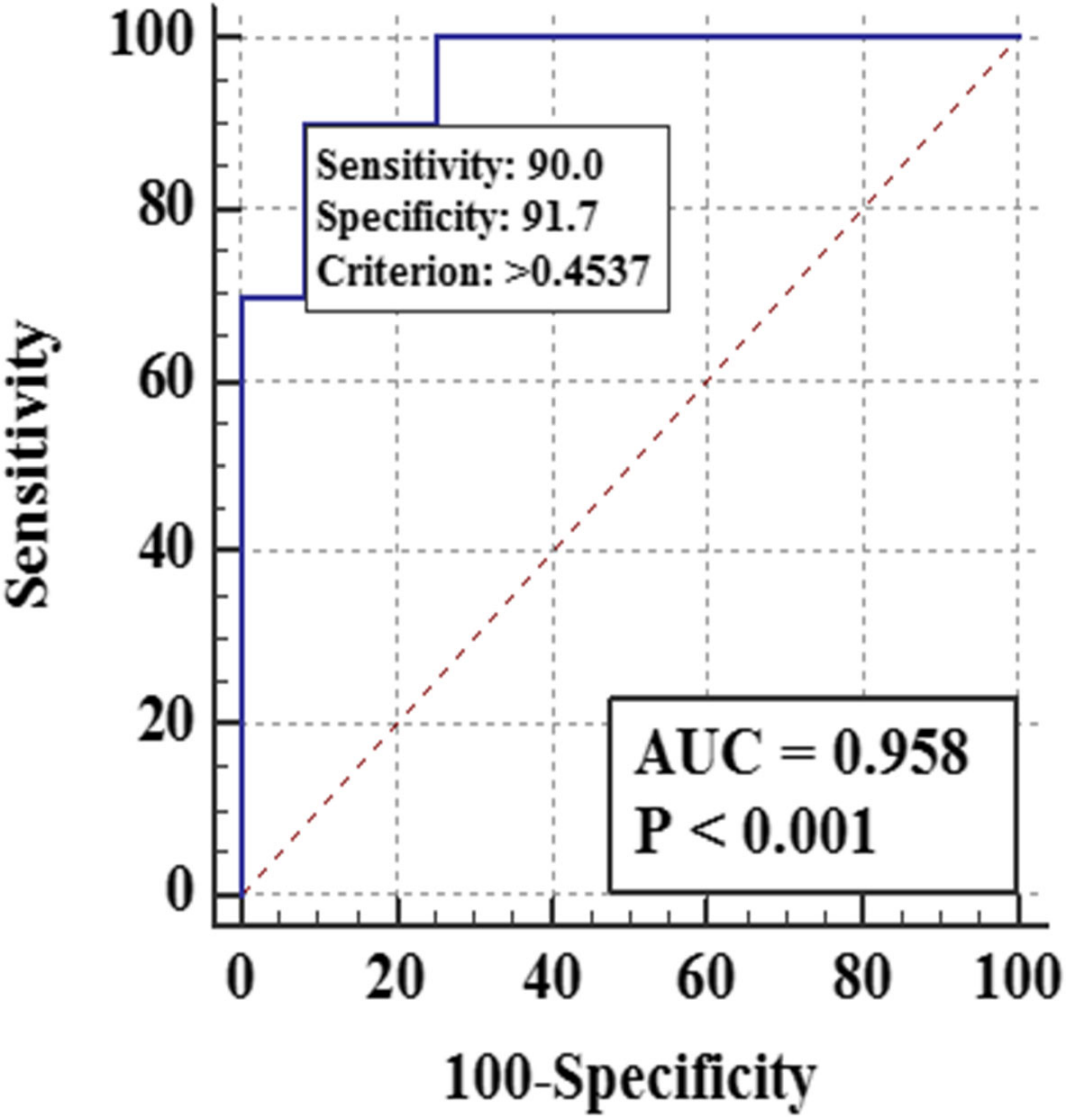
Diagnostic performance of a urinary biomarker panel for glioma detection

To evaluate the diagnostic performance of DEPs between gliomas and healthy controls, we plotted the receiver operating characteristic (ROC) curves of individual proteins and different protein patterns (Additional file: Figure S2). As shown in Fig. 4, a biomarker panel consisting of AACT, TSP4, MDHM, CALR, LEG1, and AHSG showed the best diagnostic performance for the detection of gliomas, with an area under the curve (AUC) value of 0.958. The sensitivity and specificity were 0.900 and 0.917, respectively. Compared with healthy controls, the abundance of urinary AACT increased in gliomas patients, while the abundance of urinary TSP4, MDHM, CALR, LEG1, and AHSG decreased in gliomas patients.

## Discussion

Biomarkers are measurable changes associated with physiological or pathophysiological processes. Urine accumulates systematic changes in the body and thus is an ideal sample source for biomarker research. In this study, we performed urinary biomarker discovery in glioma patients using LFQ and PRM-based targeted proteomics quantification. Our results showed that brain disorders could be reflected in the urine and that urine proteins could be used for glioma detection.

In the biomarker discovery phase, the levels of 27 urinary proteins were significantly changed after brain tumor removal. Of these DEPs, several have been reported to be associated with gliomas in previous studies, such as ANXA7, CALR, CD276, PIGR, CTSD, CRYAB, ASAH1, BGLR, and CEACAM1. For example, ANXA7 is a tumor suppressor protein. The expression of this protein is decreased in glioma tissues, and its degradation may contribute to glioma progression [18]. Moreover, loss of ANXA7 is associated with prognosis in glioblastoma patients, and ANXA7 is a strong predictor of patient outcome [33, 34]. Additionally, lower CALR levels have been observed in glioma tissues than in normal brain tissues, and CALR expression is correlated with glioma grade and patient survival [32]. CD276 antigen has been reported to be upregulated in high-grade glioma. The expression of this protein correlates with malignancy grade in gliomas and with poor patient survival [22, 35]. Additionally, expression of PIGR has been identified as a novel predictor of poor glioma patient prognosis after surgical resection [29]. CTSD has been identified as an important protein related to glioma invasion [30]. Biomarkers can be used to predict the prognosis and evaluate the treatment response of glioma patients [36]. These DEPs have potential clinical applications for aiding in the initial diagnosis of gliomas, early recognition of tumor recurrence and monitoring of treatment efficacy. As urinary proteome could be affected by some factors, such as gender, aging, exercise, and smoking. Therefore, the self-controlled method is suitable for clinical study and meets the requirements of urine biomarker research to remove interference.

After functional analysis of DEPs identified by LFQ, several important biological processes were significantly enriched, such as autophagy and angiogenesis. Autophagy is involved in tumorigenesis, and it is reportedly enhanced in glioma compared to normal brain tissue [21]. Moreover, blockade of autophagy has been proposed as an alternative therapeutic option for gliomas. In our study, five differential proteins involved in the autophagy process were significantly changed, including CTSD, NAPSA, LAMP1, LAMP2, and ANXA7. In addition, gliomas are characterized by abundant angiogenesis, and glioma progression is accompanied by extensive neovascularization [37]. Antiangiogenic therapy is also a promising approach to treat glioma and several anti-angiogenic agents have been used for target treatment of glioma [38, 39]. Several of the DEPs identified in this study are involved in angiogenesis, such as CEACAM1, AMPE, AMPN, and PCP. Thus, these proteins might be potential therapeutic targets for gliomas.

In the biomarker validation phase, some promising proteins were used for PRM-based targeted quantification. It was observed that urinary AACT and GELS were significantly upregulated in glioma patients compared to healthy controls, whereas urinary TSP4, MDHM, RINI, CALR, TENA, LEG1, and AHSG were significantly downregulated in glioma patients compared to healthy controls. Interestingly, AACT, GELS, TENA, LEG1, and AHSG had been reported as potential CSF or blood biomarkers of gliomas [40–44], which indicates the validity of our results. Moreover, we found these proteins could also serve as urinary biomarkers for noninvasive detection of gliomas. To obtain the best diagnostic performance, we plotted the ROC curves of different protein combinations. ROC curve analysis showed that the combination of six urinary proteins (AACT, TSP4, MDHM, CALR, LEG1, and AHSG) can effectively discriminate the gliomas patients from healthy controls with the AUC of 0.952. Interestingly, AACT, LEG1, and AHSG are blood or CSF biomarkers of gliomas [40, 43, 44], and CALR is correlated with glioma grade and patient survival [32].

Compared to other bodily fluids (CSF and blood), urine has been largely ignored during biomarker discovery for brain diseases. The reason may be that urine is located far from the brain, which could raise doubt as to whether pathological changes in the brains will be detected in the urine. Moreover, investigations of gliomas by using urinary proteomics have been rarely reported. In this study, using LFQ and PRM proteome quantification, we identified candidate urinary protein biomarkers with the potential to detect gliomas. Our results also suggested that brain disorders could be reflected in human urine and that urine proteins could be used to distinguish different brain diseases. However, a larger number of clinical urine samples from multi-center patients are needed to verify the specific protein pattern as biomarkers for gliomas detection.

As urine can be collected noninvasively, it is easy to repeatedly collect urine sample from the same individual for longitudinal studies. Thus, in future studies, urine samples can be collected at multiple time points for early diagnosis, early recognition of tumor recurrence, or monitoring of therapeutic efficacy in glioma patients.

## Conclusions

This is the first application of proteomics in urine samples of gliomas patients. Our results show promise for the development of urine biomarkers for gliomas and will also provide clues for future biomarker studies involving brain diseases.

## Supplementary information

**Additional file 1: Figure S1.** MRI of the brain before and after tumor resection in a glioma patient. (A) MRI for the tumor and (B) after tumor removal. **Figure S2.** ROC curves of individual DEP and different protein combinations. **Table S1.** Candidate protein biomarkers of glioma selected from the literature for PRM-based targeted validation.

## Data Availability

The datasets generated and/or analyzed during the current study are available in the figshare repository, [https://figshare.com/s/4fafe79329ee22424887].

## Abbreviations

**LFQ**: Label-free proteome quantitation
**PRM**: Parallel reaction monitoring
**ROC**: Receiver operating characteristic curve
**AUC**: Area under the curve
**AACT**: Alpha-1-antichymotrypsin
**TSP4**: Thrombospondin-4
**MDHM**: Malate dehydrogenase, mitochondrial
**CALR**: Calreticulin
**LEG1**: Galectin-1
**AHSG**: Alpha-2-HS-glycoprotein
