# Supplementary material for: Urinary biomarker discovery in gliomas using mass spectrometry-based clinical proteomics
Source: Chin Neurosurg J. 2020 Apr 14;6:11. doi: 10.1186/s41016-020-00190-5 (PMC7398313; doi:10.1186/s41016-020-00190-5)
Supplement: Supplementary file 1 — Additional file 1: Figure S1. MRI of the brain before and after tumor resection in a glioma patient. (A) MRI for the tumor and (B) after tumor removal. Figure S2. ROC curves of individual DEP and different protein combinations. Table S1. Candidate protein biomarkers of glioma selected from the literature for PRM-based targeted validation. [file 41016_2020_190_MOESM1_ESM.docx]

Supplementary

**Figure S1**. MRI of the brain before and after tumor resection in a glioma patient. (A) MRI for the tumor and (B) after tumor removal.

**Table S1.** Candidate protein biomarkers of glioma selected from the literature for PRM-based targeted validation.

**
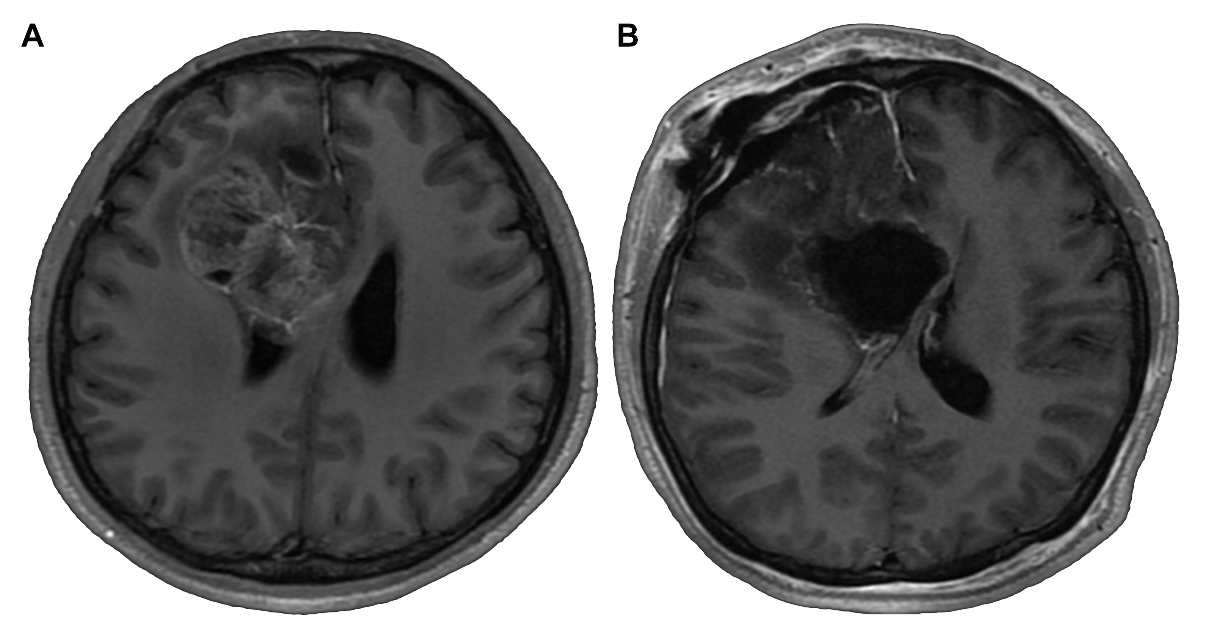
**

**Figure S1.** MRI of the brain before and after tumor resection in a glioma patient. (A) MRI for the tumor and (B) after tumor removal.

**Figure S2. ROC curves of individual DEP and different protein combinations.**

**
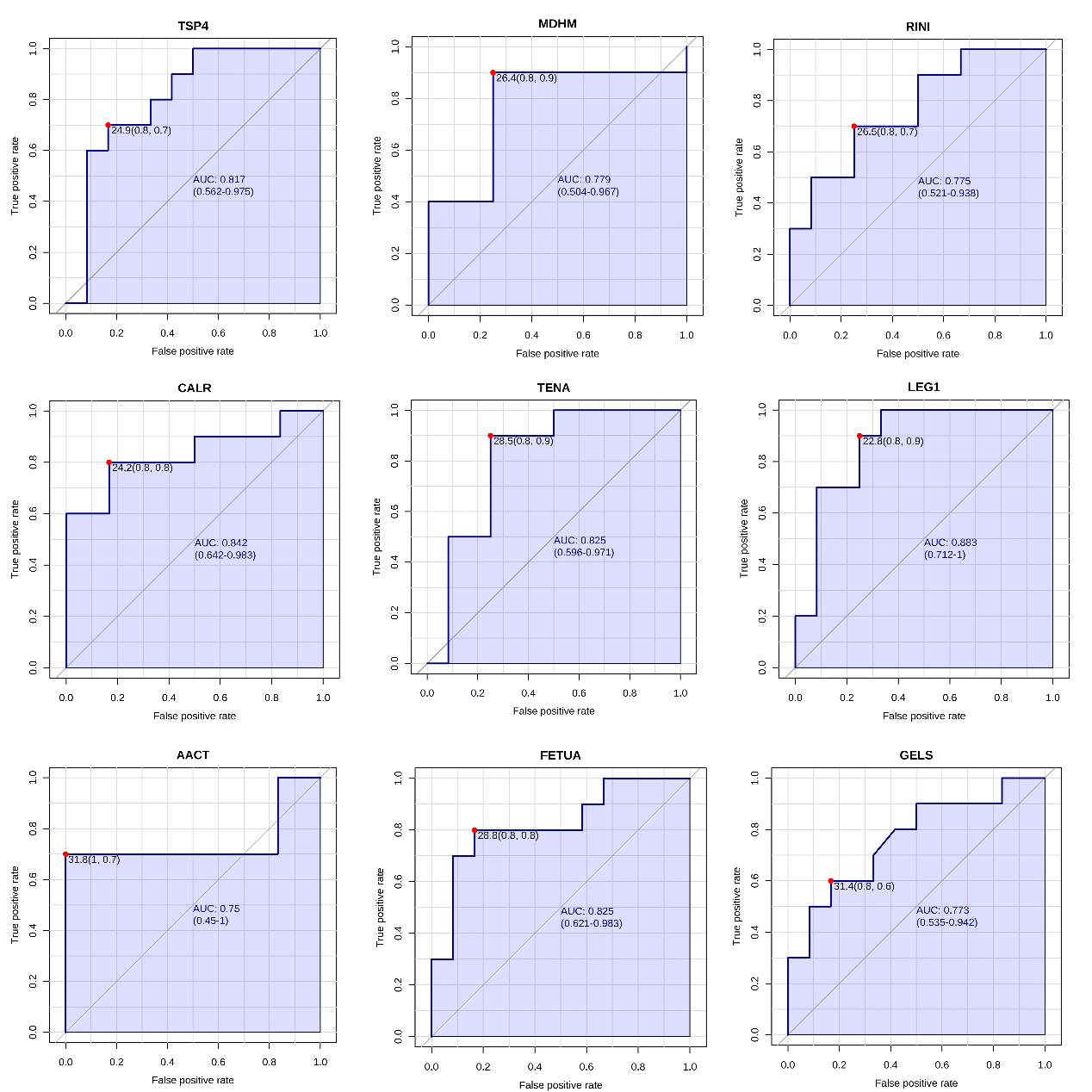
**

AUC values of different protein combination:

| **Protein combination** | **AUC value** |
| --- | --- |
| TSP4, MDHM, RINI, CALR, TENA, LEG1, AACT, AHSG and GELS | 0.927 |
| TSP4, MDHM, RINI, CALR, TENA, LEG1, AHSG and GELS | 0.923 |
| TSP4, MDHM, RINI, CALR, TENA, LEG1, AHSG | 0.929 |
| TSP4, MDHM, CALR, TENA, LEG1, AHSG | 0.939 |
| TSP4, CALR, TENA, AHSG | 0.921 |
| TSP4, CALR, LEG1, AHSG | 0.926 |
| TSP4, CALR, LEG1, AHSG, MDHM | 0.943 |
| TSP4, CALR, LEG1, AHSG, MDHM, AACT | 0.958 |

**Table S1. Protein candidates of glioma in the literature for PRM targeted validation.**

| **Uniprot** | **Protein name** | **Biomarkers**† | **Ref.** |
| --- | --- | --- | --- |
| P10451 | Osteopontin (OPN) | CSF | [1] |
| P18065 | Insulin-like growth factor-binding protein 2 (IGFBP2) | CSF; Blood | [2,3] |
| P14780 | Matrix metalloproteinase-9 (MMP9) | Blood | [4,5] |
| P15692 | Vascular endothelial growth factor A (VEGF) | CSF; Blood | [6,7] |
| O15123 | Angiopoietin-2 (Ang-2) | Blood | [8] |
| P04085 | Platelet derived growth factor (PDGF) | Blood | [7,9] |
| P29474 | Endothelial nitric oxide synthase (eNOS) | Blood | [10] |
| P07996 | Thrombosponin-1 (TSP1) | Blood | [11] |
| P24821 | Tenascin (TNC) | CSF | [12] |
| P14136 | Glial fibrillary acidic protein (GFAP) | Blood | [13,14] |
| P06731 | Carcinoembryonal antigen (CEA) | CSF, Blood | [15,16] |
| P36222 | Chitinase-3-like protein 1 (YKL-40) | Blood | [5,17] |
| P09382 | Galectin-1 (LEG1) | Blood | [18] |
| P01138 | Nerve growth factor (NGF) | CSF | [19] |
| P14174 | Macrophage migration inhibitory factor (MIF) | CSF | [20] |
| P02766 | Transthyretin (TTHY) | CSF | [21] |
| P01011 | Alpha-1-antichymotrypsin (AACT) | CSF | [21] |
| P13591 | Neural cell adhesion molecule (NCAM) | Blood | [22] |
| P00533 | Epidermal growth factor receptor (EGFR) | Blood | [23] |
| P02765 | Alpha-2-HS-glycoprotein (AHSG) | Blood | [24] |
| P05121 | Plasminogen activator inhibitor 1 (PAI-1) | Blood | [25] |
| O75882 | Attractin (ATRN) | CSF | [26] |
| P06396 | Gelsolin (GSN) | CSF | [27] |
| O75888 | Tumor necrosis factor ligand superfamily member 13 (APRIL) | Blood | [28] |
| P43490 | Nicotinamide phosphoribosyltransferase (PBEF1) | Blood | [29] |
| P35243 | Recoverin (RCVRN) | Blood | [30] |
| P09038 | Fibroblast growth factor 2 (FGFB) | CSF; Blood | [6,31] |

†Candidate biomarkers identified from blood or CSF of glioma patients.

**References:**

1. Ellert-Miklaszewska A, Wisniewski P, Kijewska M, et al. Tumour-processed osteopontin and lactadherin drive the protumorigenic reprogramming of microglia and glioma progression. Oncogene 2016;35:6366-77.

2. Li Y, Jiang T, Zhang J, et al. Elevated serum antibodies against insulin-like growth factor-binding protein-2 allow detecting early-stage cancers: evidences from glioma and colorectal carcinoma studies. Ann Oncol 2012;23:2415-22.

3. Lin Y, Jiang T, Zhou K, et al. Plasma IGFBP-2 levels predict clinical outcomes of patients with high-grade gliomas. Neuro Oncol 2009;11:468-76.

4. Iwamoto FM, Hottinger AF, Karimi S, et al. Longitudinal prospective study of matrix metalloproteinase-9 as a serum marker in gliomas. J Neurooncol 2011;105:607-12.

5. Hormigo A, Gu B, Karimi S, et al. YKL-40 and matrix metalloproteinase-9 as potential serum biomarkers for patients with high-grade gliomas. Clin Cancer Res 2006;12:5698-704.

6. Peles E, Lidar Z, Simon AJ, et al. Angiogenic factors in the cerebrospinal fluid of patients with astrocytic brain tumors. Neurosurgery 2004;55:562-7; discussion 7-8.

7. Ilhan A, Gartner W, Neziri D, et al. Angiogenic factors in plasma of brain tumour patients. Anticancer Res 2009;29:731-6.

8. Batchelor TT, Duda DG, di Tomaso E, et al. Phase II study of cediranib, an oral pan-vascular endothelial growth factor receptor tyrosine kinase inhibitor, in patients with recurrent glioblastoma. J Clin Oncol 2010;28:2817-23.

9. Chinnaiyan P, Chowdhary S, Potthast L, et al. Phase I trial of vorinostat combined with bevacizumab and CPT-11 in recurrent glioblastoma. Neuro Oncol 2012;14:93-100.

10. Zheng PP, Hop WC, Luider TM, et al. Increased levels of circulating endothelial progenitor cells and circulating endothelial nitric oxide synthase in patients with gliomas. Ann Neurol 2007;62:40-8.

11. Elstner A, Stockhammer F, Nguyen-Dobinsky TN, et al. Identification of diagnostic serum protein profiles of glioblastoma patients. J Neurooncol 2011;102:71-80.

12. Yoshida J, Wakabayashi T, Okamoto S, et al. Tenascin in cerebrospinal fluid is a useful biomarker for the diagnosis of brain tumour. Journal of Neurology, Neurosurgery &amp; Psychiatry 1994;57:1212-5.

13. Kiviniemi A, Gardberg M, Frantzen J, et al. Serum levels of GFAP and EGFR in primary and recurrent high-grade gliomas: correlation to tumor volume, molecular markers, and progression-free survival. J Neurooncol 2015;124:237-45.

14. Brommeland T, Rosengren L, Fridlund S, et al. Serum levels of glial fibrillary acidic protein correlate to tumour volume of high-grade gliomas. Acta Neurol Scand 2007;116:380-4.

15. Rombos A, Evangelopoulu-Katsiri E, Mariatos P, et al. Cerebrospinal fluid carcinoembryonic antigen and alphafetoprotein in patients with central nervous system neoplasia. Acta Neurol Scand 1988;77:440-4.

16. Batabyal SK, Ghosh B, Sengupta S, et al. Cerebrospinal fluid and serum carcinoembryonic antigen in brain tumors. Neoplasma 2003;50:377-9.

17. Bernardi D, Padoan A, Ballin A, et al. Serum YKL-40 following resection for cerebral glioblastoma. J Neurooncol 2012;107:299-305.

18. Verschuere T, Van Woensel M, Fieuws S, et al. Altered galectin-1 serum levels in patients diagnosed with high-grade glioma. J Neurooncol 2013;115:9-17.

19. Li QY, Yang Y, Zhang Y, et al. Nerve growth factor expression in astrocytoma and cerebrospinal fluid: a new biomarker for prognosis of astrocytoma. Chin Med J (Engl) 2011;124:2222-7.

20. Mittelbronn M, Platten M, Zeiner P, et al. Macrophage migration inhibitory factor (MIF) expression in human malignant gliomas contributes to immune escape and tumour progression. Acta Neuropathol 2011;122:353-65.

21. Schuhmann MU, Zucht HD, Nassimi R, et al. Peptide screening of cerebrospinal fluid in patients with glioblastoma multiforme. Eur J Surg Oncol 2010;36:201-7.

22. Todaro L, Christiansen S, Varela M, et al. Alteration of serum and tumoral neural cell adhesion molecule (NCAM) isoforms in patients with brain tumors. J Neurooncol 2007;83:135-44.

23. Quaranta M, Divella R, Daniele A, et al. Epidermal growth factor receptor serum levels and prognostic value in malignant gliomas. Tumori 2007;93:275-80.

24. Petrik V, Saadoun S, Loosemore A, et al. Serum alpha 2-HS glycoprotein predicts survival in patients with glioblastoma. Clin Chem 2008;54:713-22.

25. Iwadate Y, Hayama M, Adachi A, et al. High serum level of plasminogen activator inhibitor-1 predicts histological grade of intracerebral gliomas. Anticancer Res 2008;28:415-8.

26. Khwaja FW, Duke-Cohan JS, Brat DJ, et al. Attractin is elevated in the cerebrospinal fluid of patients with malignant astrocytoma and mediates glioma cell migration. Clin Cancer Res 2006;12:6331-6.

27. Ohnishi M, Matsumoto T, Nagashio R, et al. Proteomics of tumor-specific proteins in cerebrospinal fluid of patients with astrocytoma: usefulness of gelsolin protein. Pathol Int 2009;59:797-803.

28. Ilzecka J, Ilzecki M. APRIL is increased in serum of patients with brain glioblastoma multiforme. Eur Cytokine Netw 2006;17:276-80.

29. Reddy PS, Umesh S, Thota B, et al. PBEF1/NAmPRTase/Visfatin: a potential malignant astrocytoma/glioblastoma serum marker with prognostic value. Cancer Biol Ther 2008;7:663-8.

30. Sampath P, Weaver CE, Sungarian A, et al. Cerebrospinal fluid (vascular endothelial growth factor) and serologic (recoverin) tumor markers for malignant glioma. Cancer Control 2004;11:174-80.

31. Fine HA, Figg WD, Jaeckle K, et al. Phase II trial of the antiangiogenic agent thalidomide in patients with recurrent high-grade gliomas. J Clin Oncol 2000;18:708-15.
